# Supplementary figures and images for: Genomic signatures defining responsiveness to allopurinol and combination therapy for lung cancer identified by systems therapeutics analyses
Source: Mol Oncol. 2019 Jul 10;13(8):1725–43. doi: 10.1002/1878-0261.12521 (PMC6670022; doi:10.1002/1878-0261.12521)

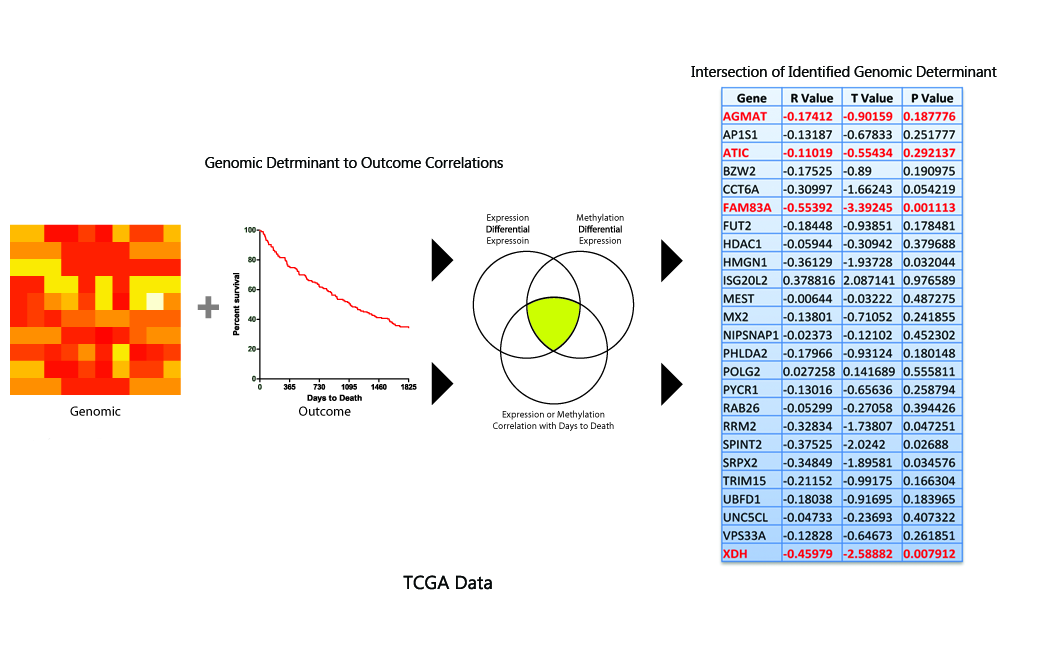

Supplement: Supplementary file 1 — Fig. S1. The pipeline used to analyze TCGA data combining molecular alterations and clinical outcome to find new targets that are determined by the clinical outcomes in patients. [file MOL2-13-1725-s001.tif]

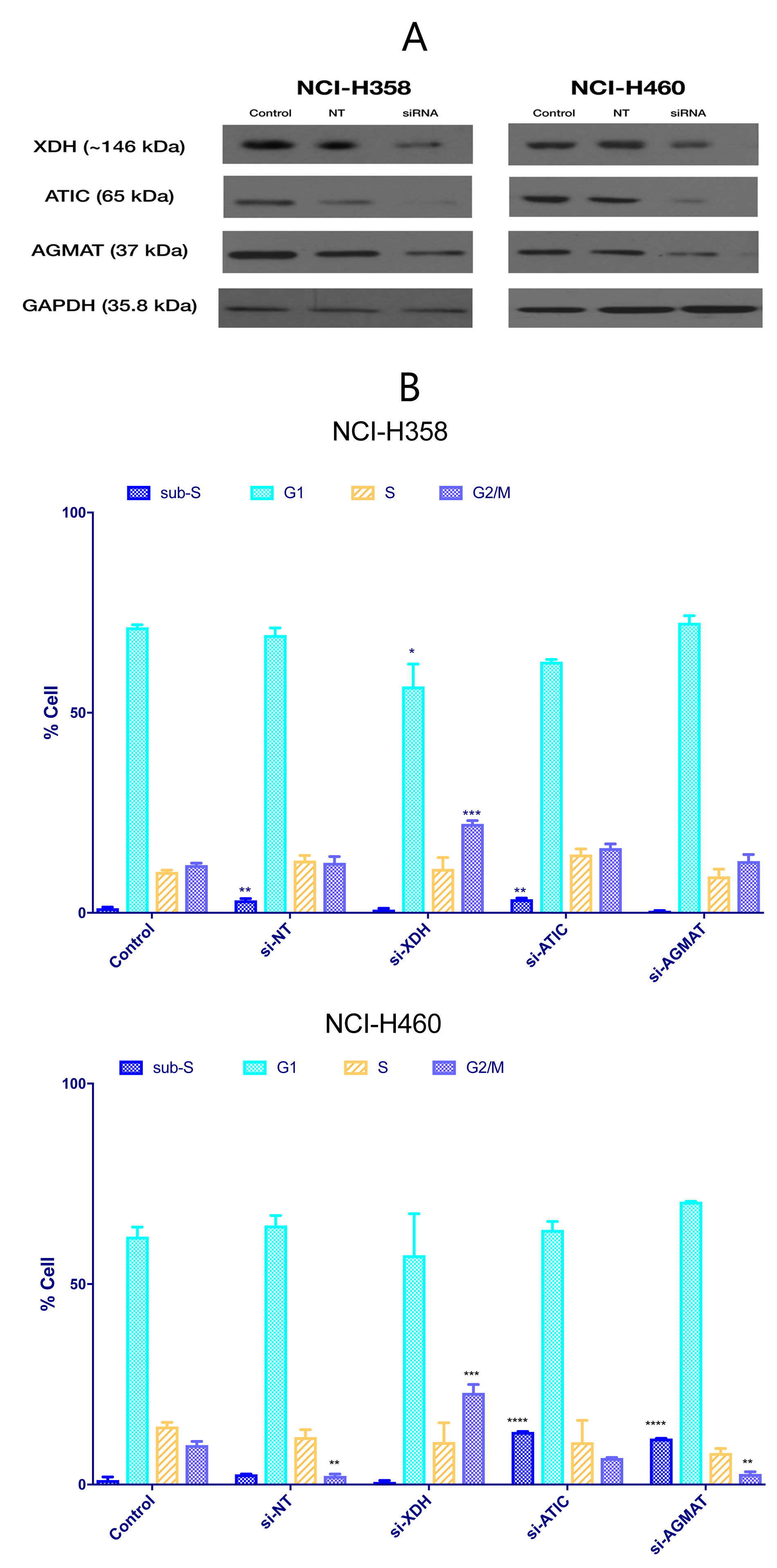

Supplement: Supplementary file 2 — Fig. S2. (A) Western blots showing the protein levels of three selected gene targets after siRNA knockdown in two of the 12 cell lines tested. (B) Comparison of cell cycle phases in two cell lines after knockdown of AGMT, ATIC and XDH compared to control. XDH knockdown increased cells arrested in G2/M phase (one‐way ANOVA, *P < 0.05, **P < 0.01, ***P < 0.001, ****P < 0.0001). [file MOL2-13-1725-s002.tif]

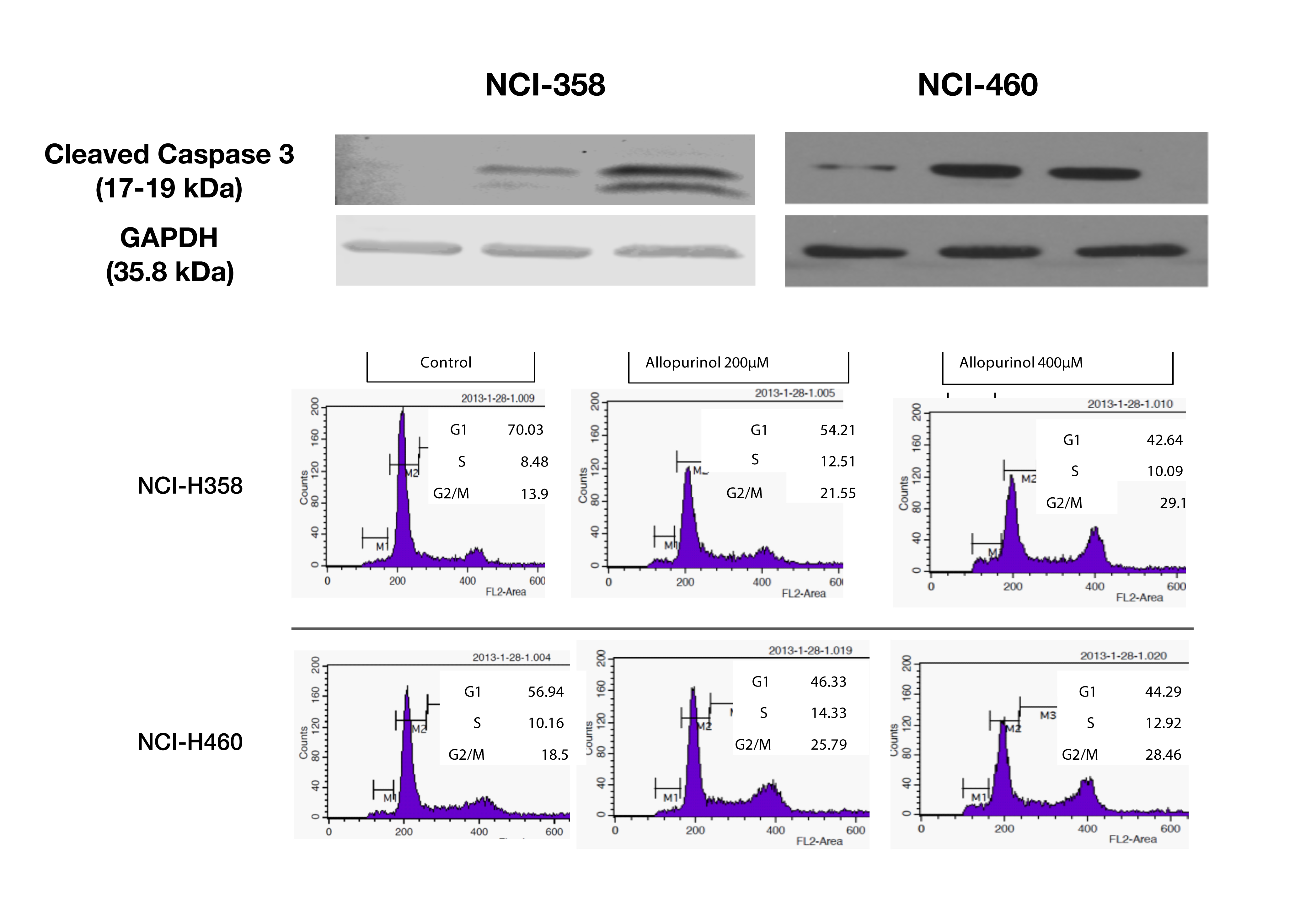

Supplement: Supplementary file 3 — Fig. S3. (A) Apoptosis induction by allopurinol shown by detection of cleaved caspase‐3 in NCI‐H358 and NCI‐H460 cell lines. (B) Compared to control‐vehicle, allopurinol arrested the cells (NCI‐H358 and NCI‐H460) in G2/M phase shown by cell cycle analysis using flow cytometry. [file MOL2-13-1725-s003.tif]

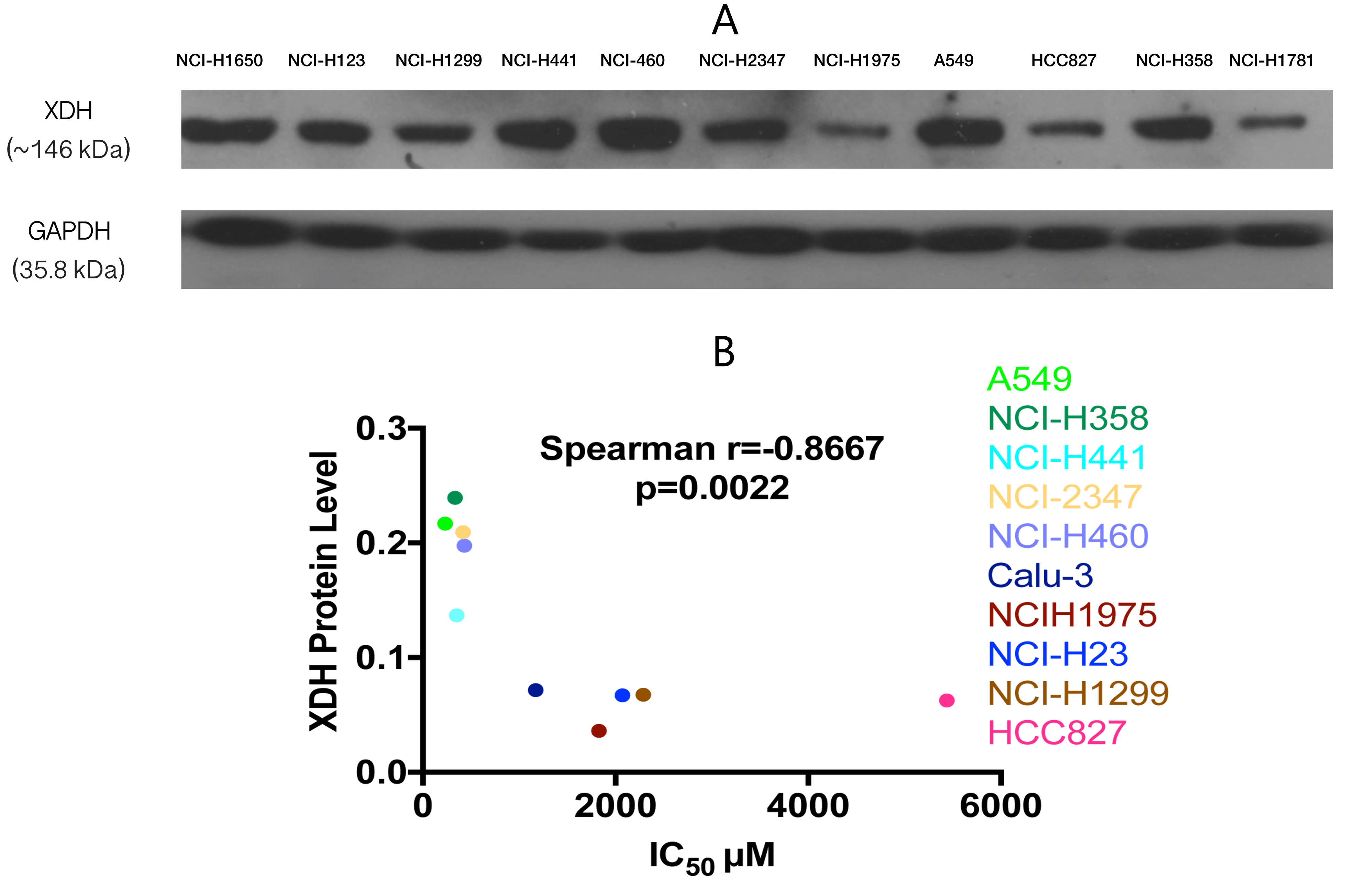

Supplement: Supplementary file 4 — Fig. S4. (A) Basal XDH protein levels in the NSCLC cell lines. (B) XDH protein levels negatively correlate with the IC50 for allopurinol in cell lines (Spearman r = −0.8667, P = 0.0022). Cell lines sensitive to allopurinol have higher levels of XDH protein indicating an addiction to XDH protein. [file MOL2-13-1725-s004.jpg]

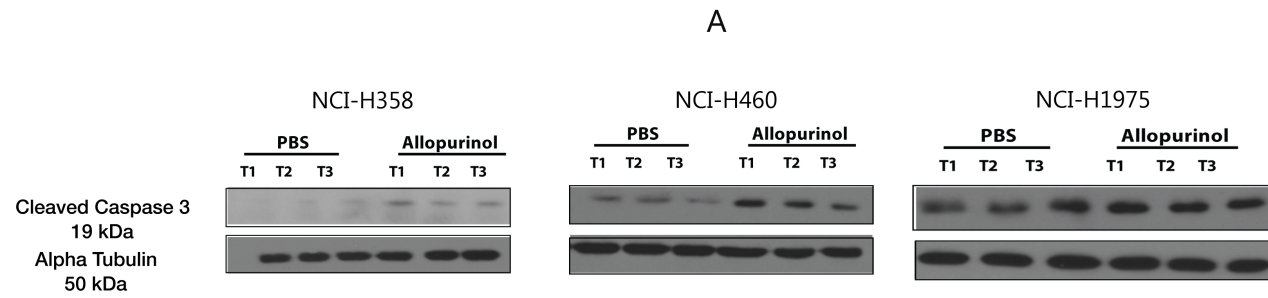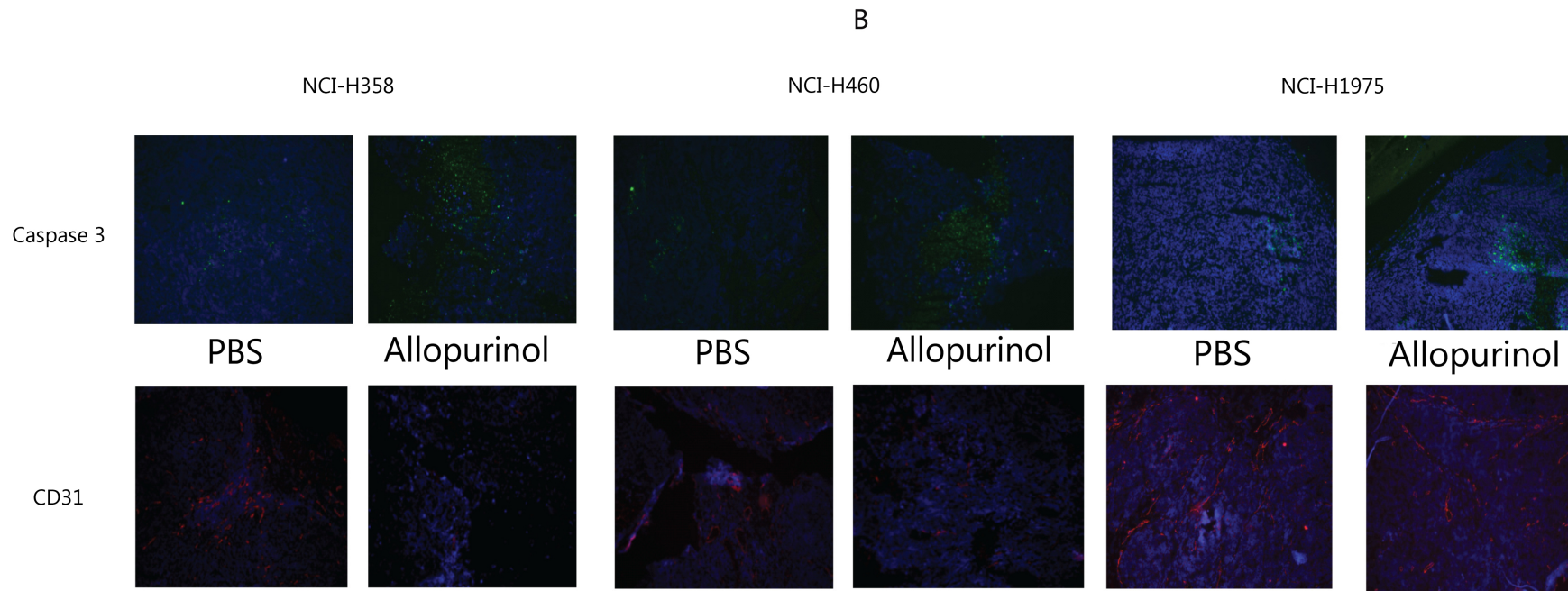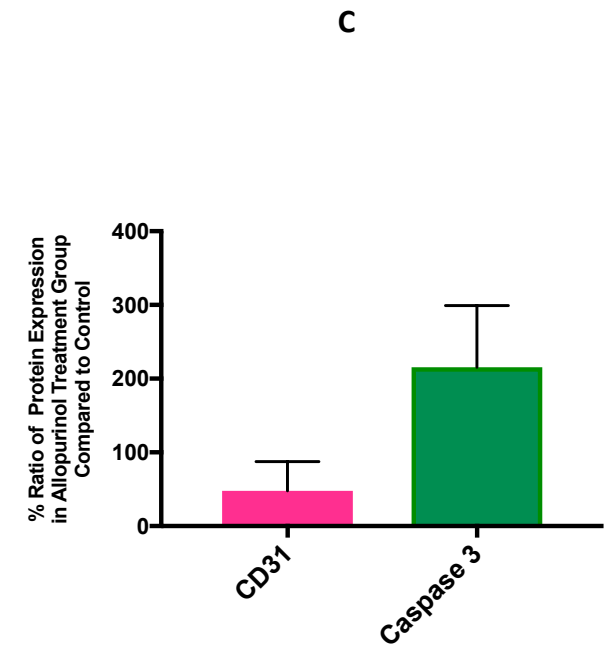

Supplement: Supplementary file 5 — Fig. S5. (A) Allopurinol‐induced apoptosis presented as the expression of cleaved caspase‐3 in xenograft models of allopurinol‐sensitive cell lines (NCI‐H358 and NCI‐H460) but not in NCI‐1975 which is allopurinol‐resistant. T1‐T3 show three different tumor samples. (B) Immunofluorescence images of xenografts from PBS and allopurinol‐treated mice. Apoptosis induced by allopurinol is indicated by cleaved caspase‐3 expression while decreased blood vessel density is indicated by CD31 expression. C) Quantification of caspase‐3 and CD31 protein expressions shown as %ratio in the all of allopurinol treatment groups (three cell lines in panel B) compared to their controls from the immunofluorescence images in panel B. [file MOL2-13-1725-s005.pdf]

NCI-H358

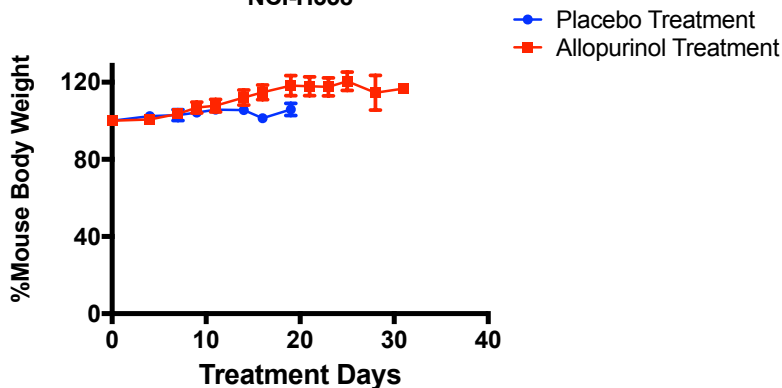

NCI-H460

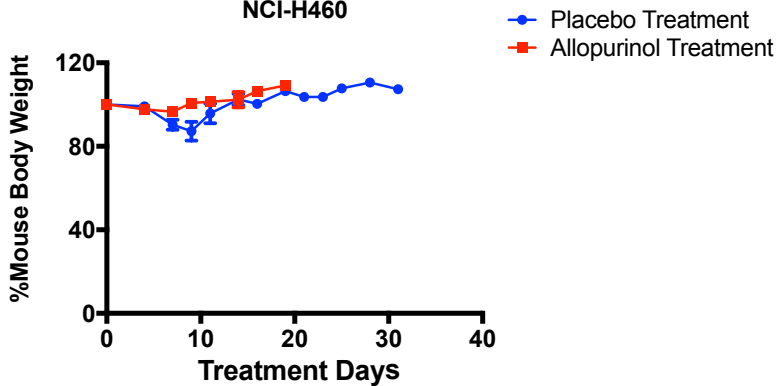

NCI-H1975

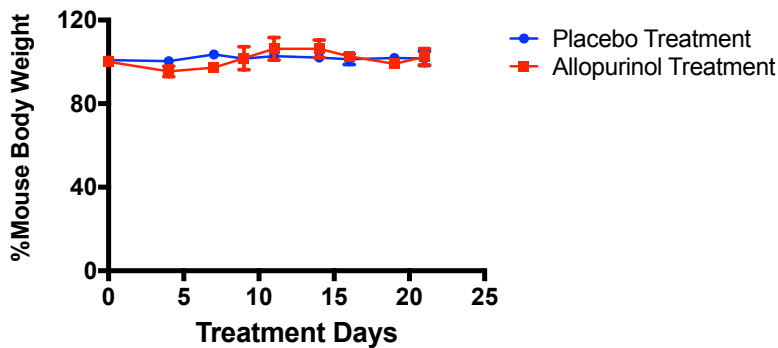

Supplement: Supplementary file 6 — Fig. S6. Changes in body weight for the xenograft models of all treatment regimens. [file MOL2-13-1725-s006.pdf]

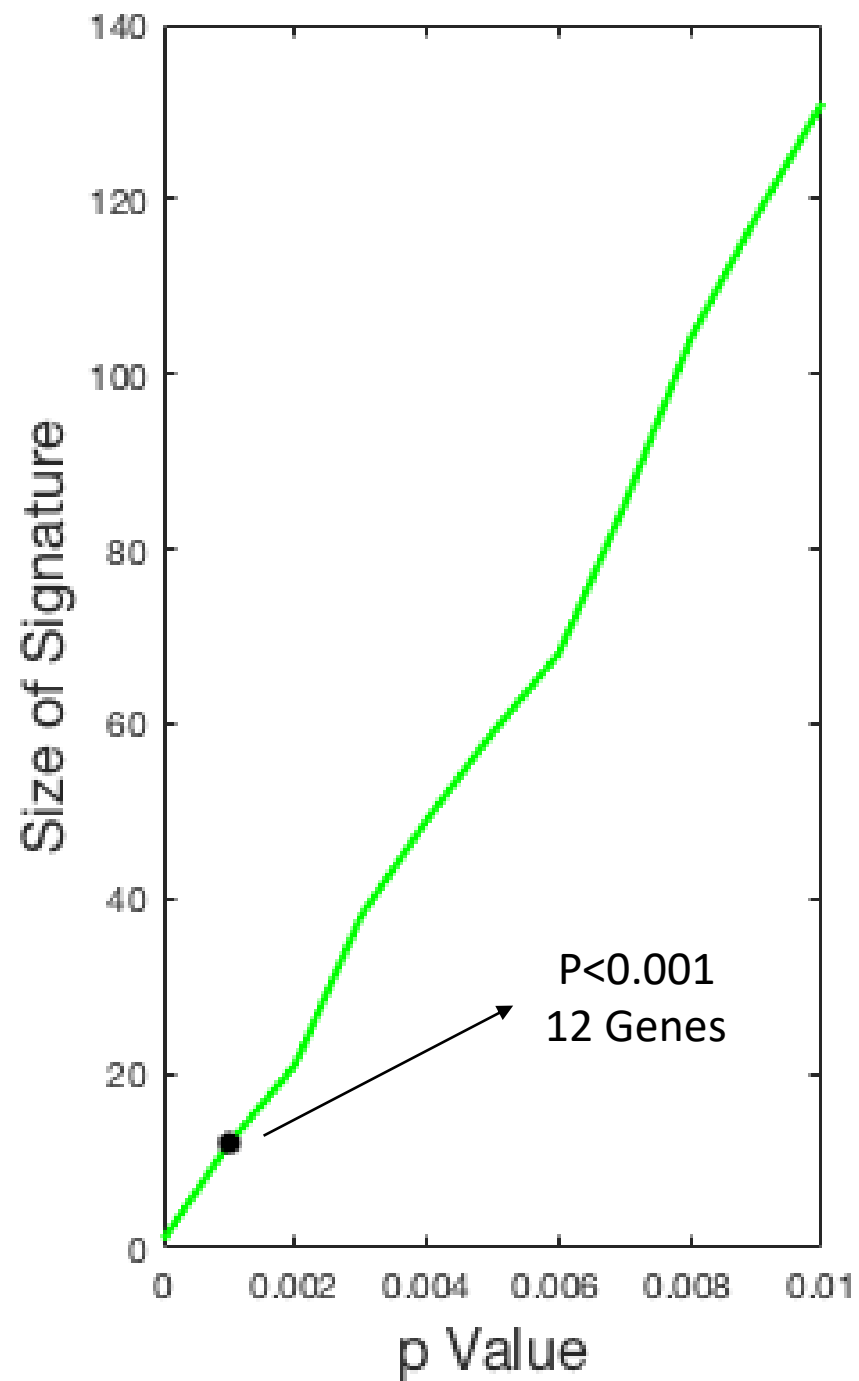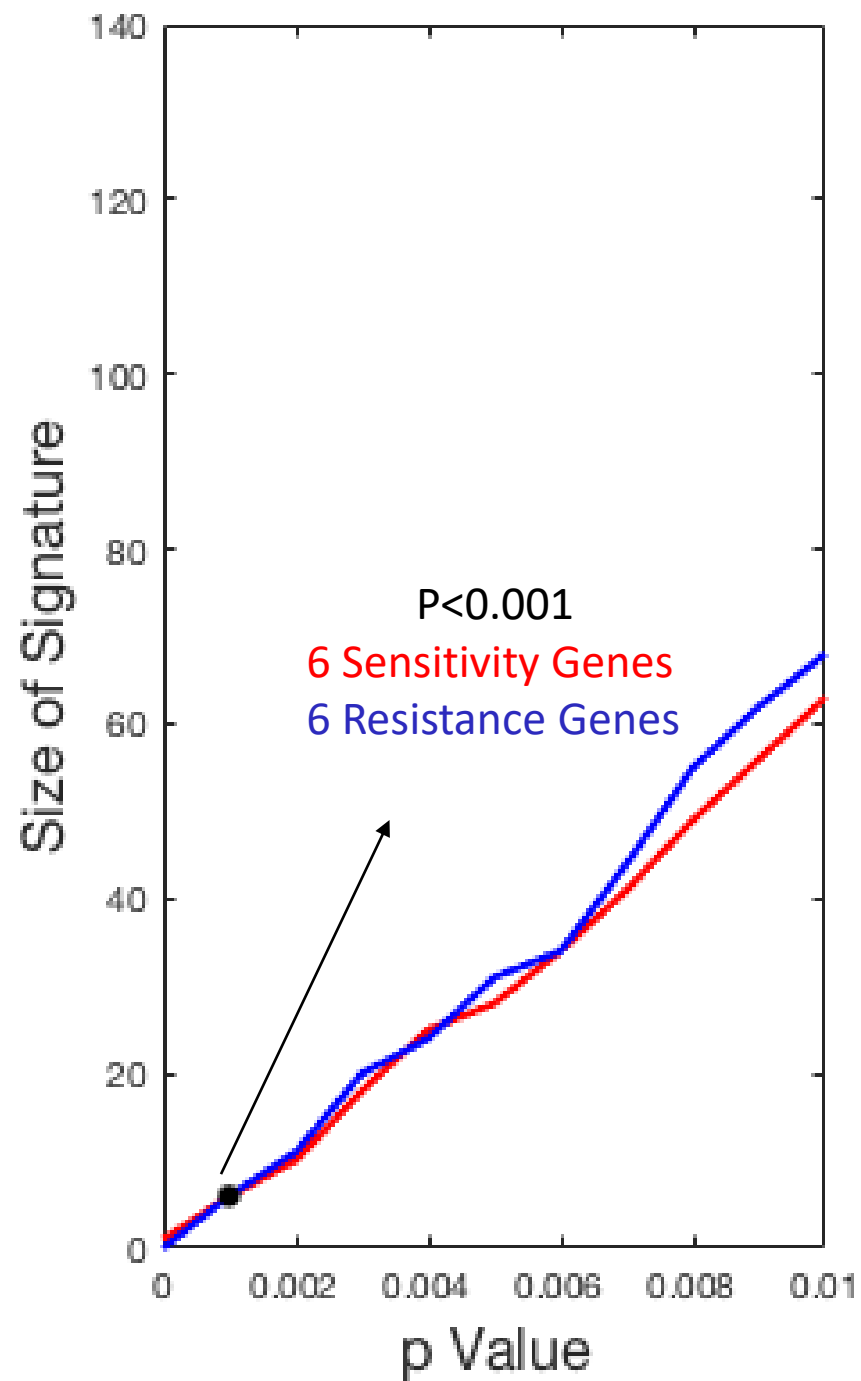

Supplement: Supplementary file 7 — Fig. S7. The number of genes in genomic signatures (genomic signature size) is determined by the stringency of statistical analysis on genomic profiles of samples (cell lines). By increasing the P value, the genomic signature size increases. Using P = 0.001 leads to a genomic signature with a size of 12 (sensitivity signature size of 6 and resistance signature size of 6). Panel A shows changes of size of genomic signature (Resistance and Sensitivity together) for different P values. Panel B shows changes of size of sensitivity genomic signature (red line) and resistance genomic signature (blue line) for different P values. [file MOL2-13-1725-s007.pdf]

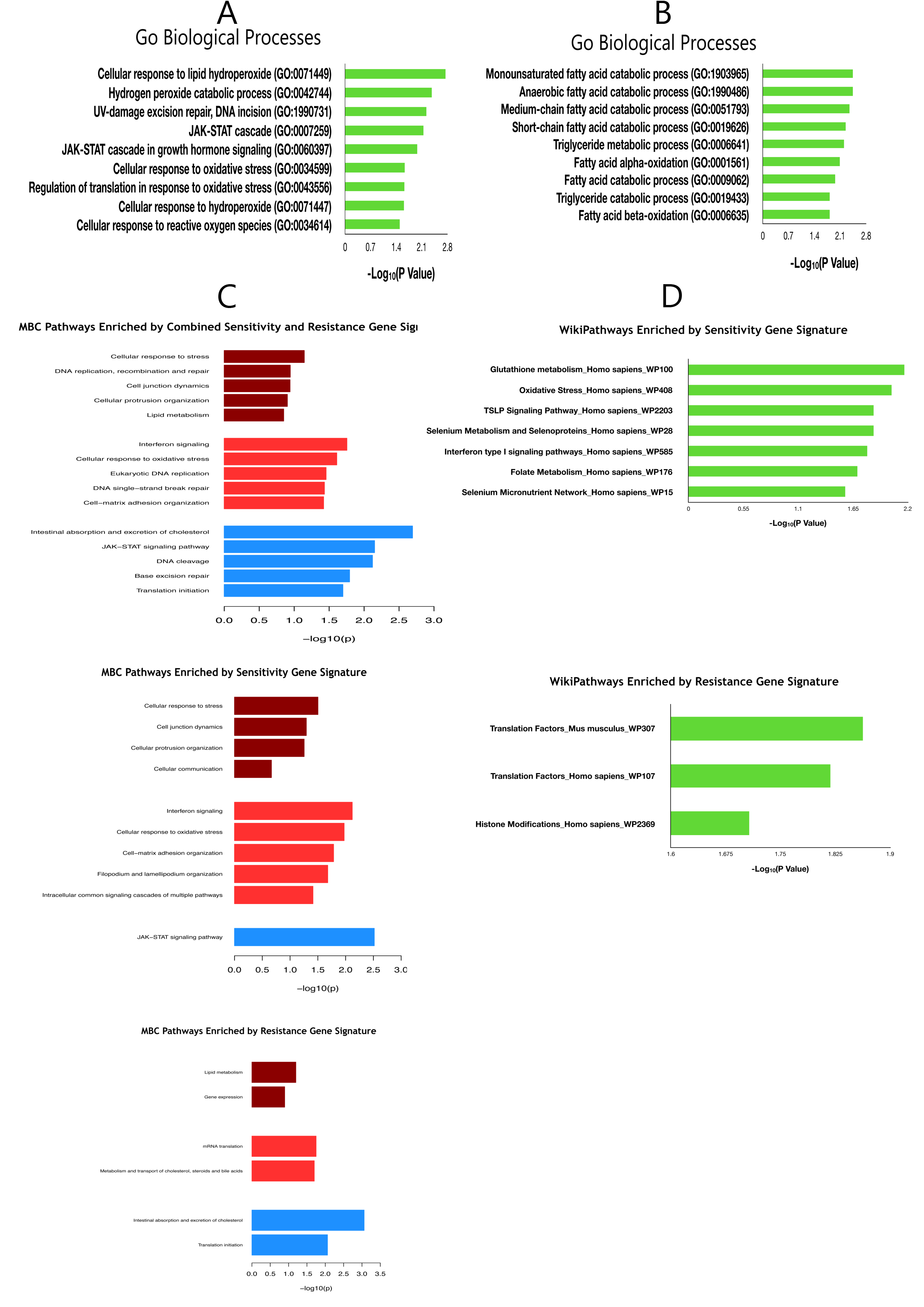

Supplement: Supplementary file 8 — Fig. S8. Gene set enrichment analysis of genomic signatures of allopurinol sensitivity and resistance using GO terms (A and B, for sensitivity genes set and resistance genes set respectively) MBC ontology (B) and WikiPathways (C). [file MOL2-13-1725-s008.tif]

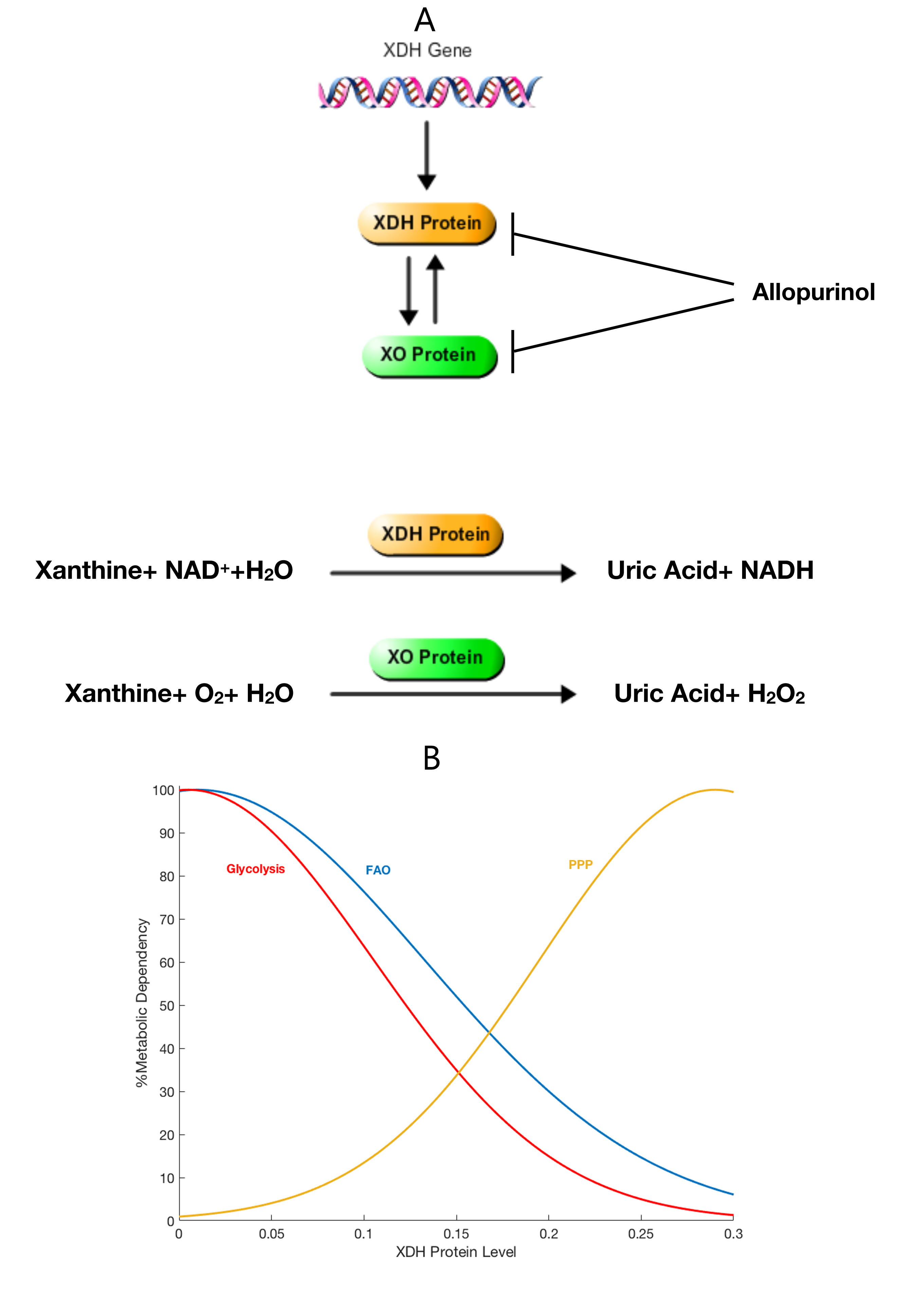

Supplement: Supplementary file 9 — Fig. S9. (A) Schematic mechanism of allopurinol and function of XDH protein in cells. (B) A mathematical phenomenological model (A Fuzzy Metabolic Switch) that can explain addiction to XDH protein in allopurinol‐sensitive cells which have higher levels of basal XDH protein. Based on the genomic signatures and enrichment analysis these cell lines can be more dependent to PPP by increasing their XDH protein level while resistant cell lines can be more dependent on Fatty Acid Oxidation (FAO) and Glycolysis. Allopurinol inhibits XDH protein leading to metabolic stress and cell death. [file MOL2-13-1725-s009.jpg]

## NCI-H1975

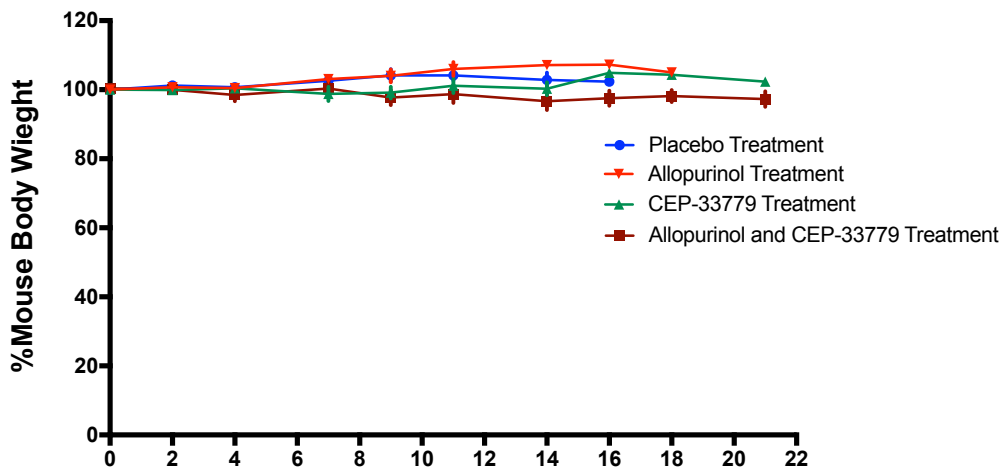

## NCI-H1650

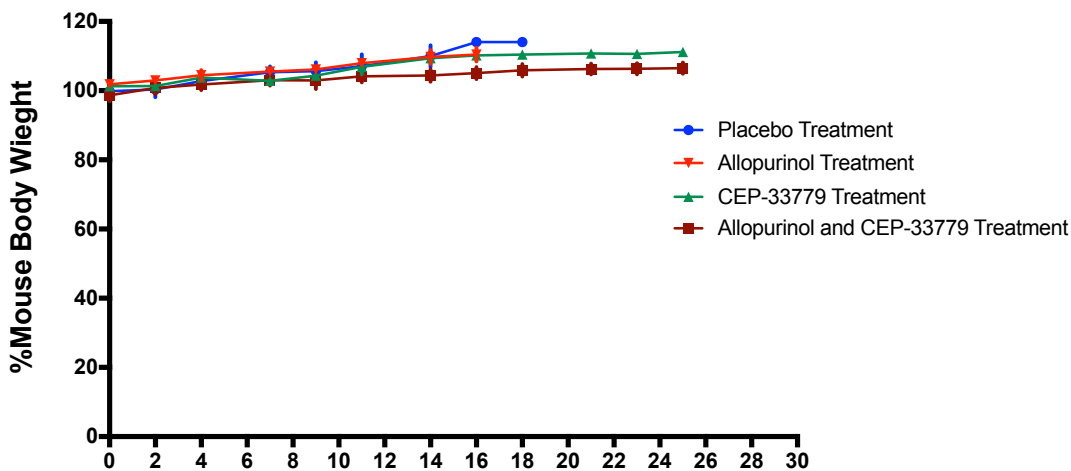

## HCC827

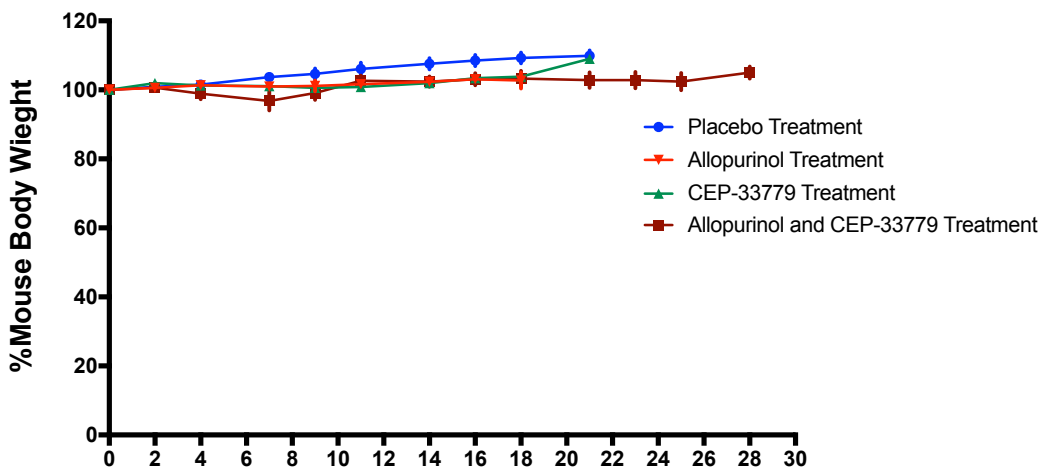

Supplement: Supplementary file 11 — Fig. S11. Changes in the body weight of mice used as xenograft models for three different cell lines to evaluate combination therapy with CEP‐33779 and allopurinol (mean ± SEM). [file MOL2-13-1725-s011.pdf]

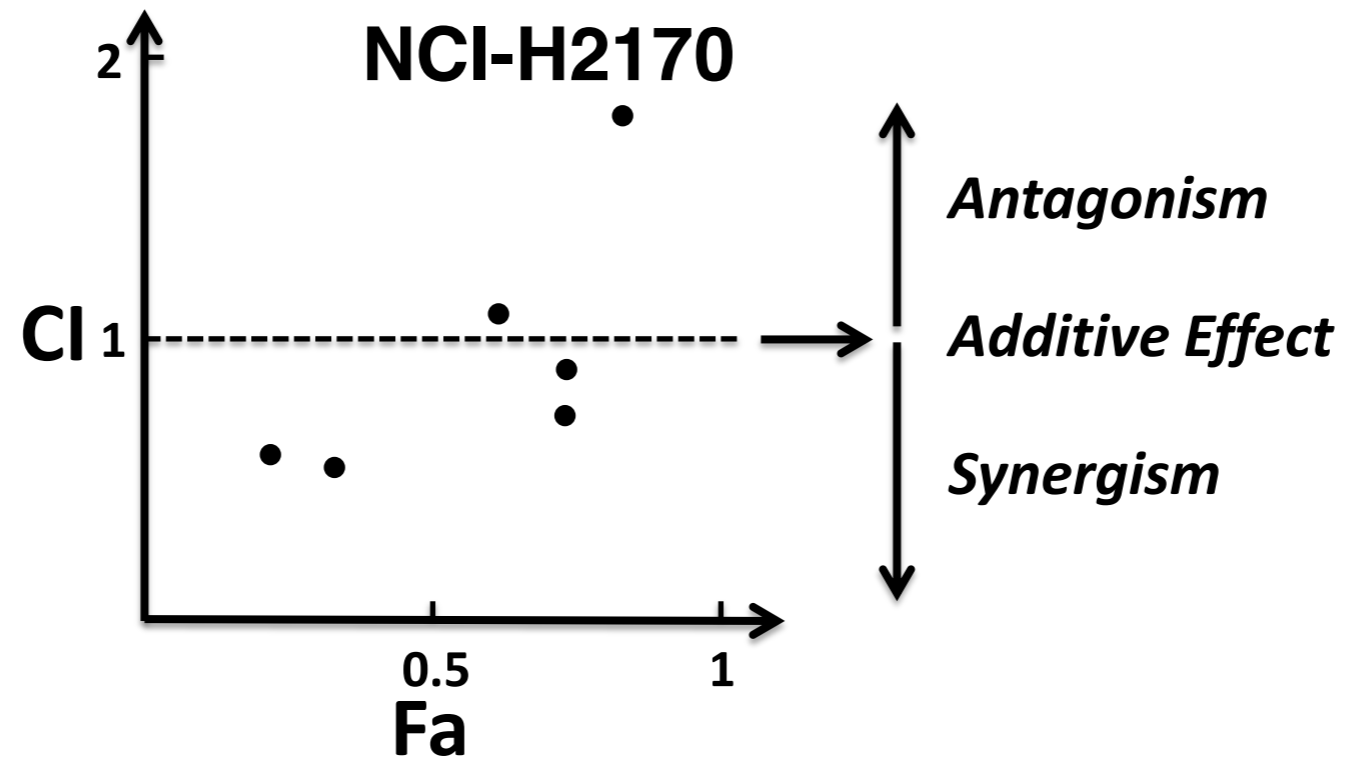

Supplement: Supplementary file 12 — Fig. S12. CI for different doses of allopurinol and CEP‐33779 in NCI‐H2170. [file MOL2-13-1725-s012.pdf]

NCI-H2106

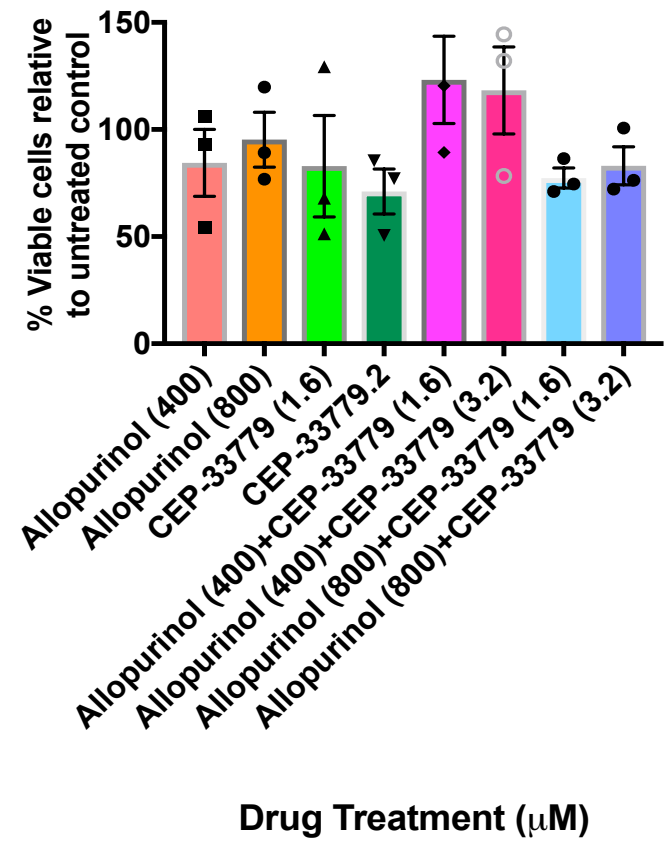

Supplement: Supplementary file 13 — Fig. S13. Effects of combination treatment with CEP‐33779 and allopurinol on cell viability of NCI‐H2106. This cell line was inactive in response to single treatments and combination treatments. [file MOL2-13-1725-s013.pdf]

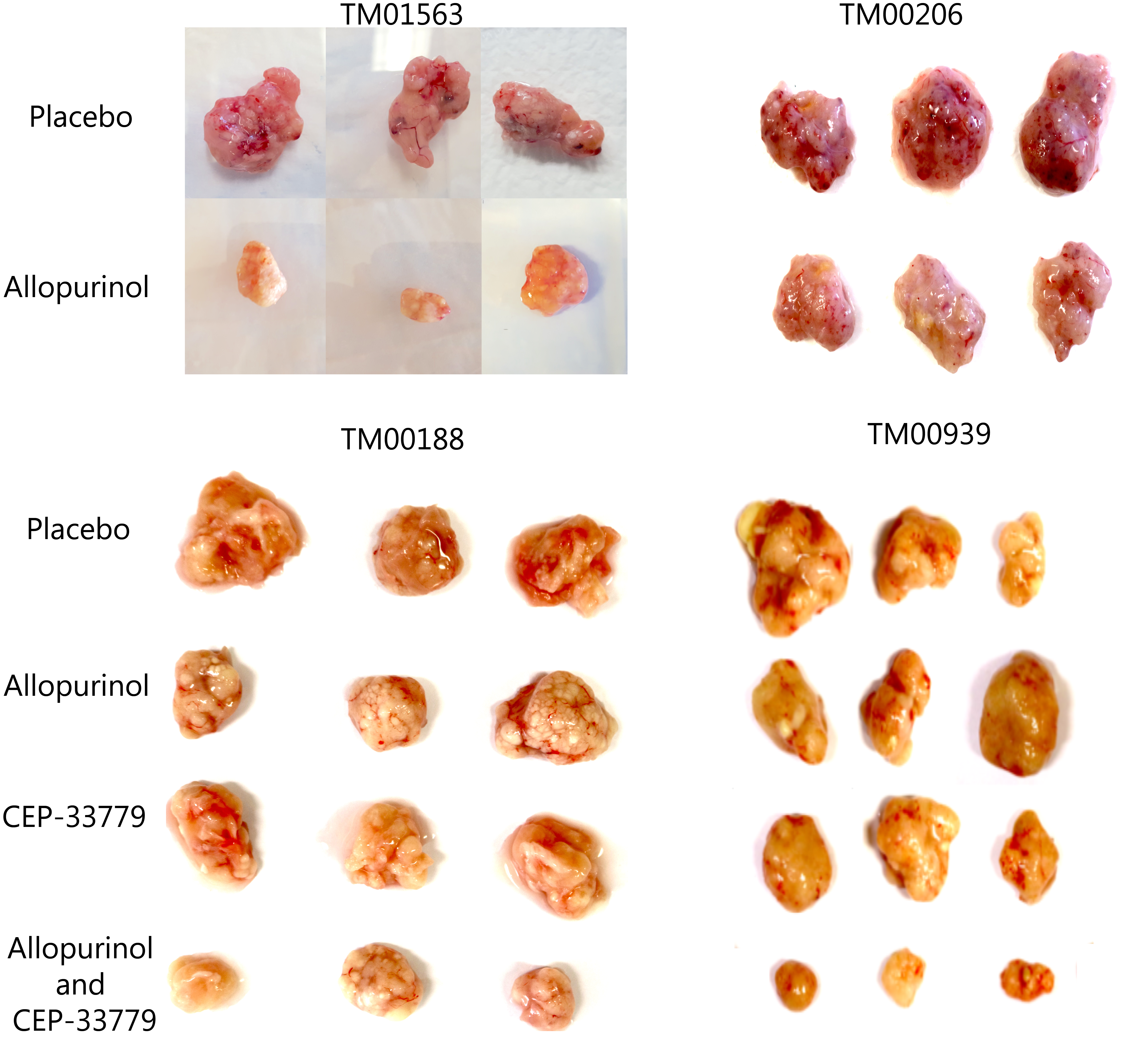

Supplement: Supplementary file 14 — Fig. S14. Images of three tumors of different treatment groups after treatment of 4 different PDX models. (Allopurinol (70 mg·kg−1 daily), CEP‐33779 (10 mg·kg−1 daily), combination therapy (Allopurinol 50 mg·kg−1 and CEP‐33779 2.5 mg·kg−1 daily) and PBS as placebo daily). [file MOL2-13-1725-s014.tif]

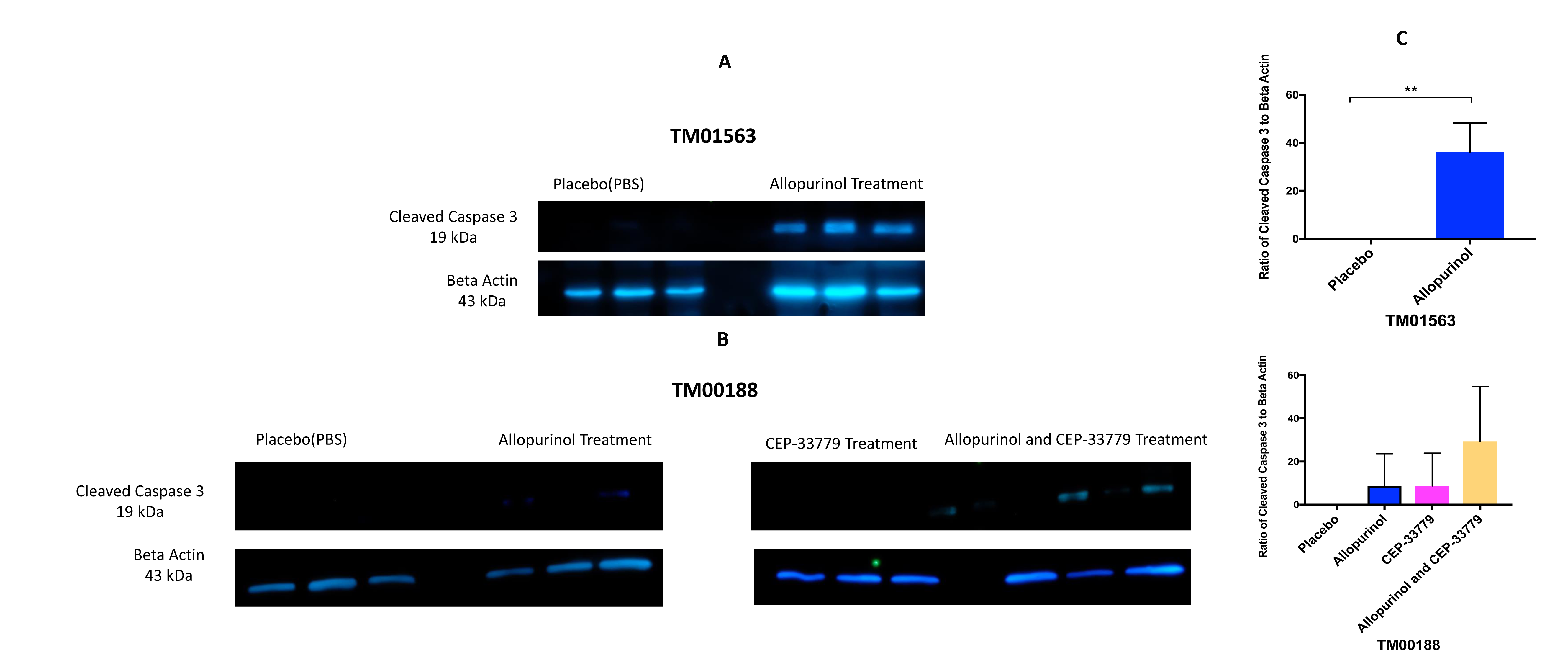

Supplement: Supplementary file 15 — Fig. S15. (A, B) Western blots of tumor samples after treatment course; apoptosis was measured by the presence of cleaved caspase‐3 in TM01563 and TM00188 models. (Allopurinol (70 mg·kg−1 daily), CEP‐33779 (10 mg·kg−1 daily), combination therapy (Allopurinol 50 mg·kg−1 and CEP‐33779 2.5 mg·kg−1 daily) and PBS as placebo daily). (C) Quantification of ratios of cleaved caspase‐3 to beta actin in western blots in panel A and B. [file MOL2-13-1725-s015.tif]

A

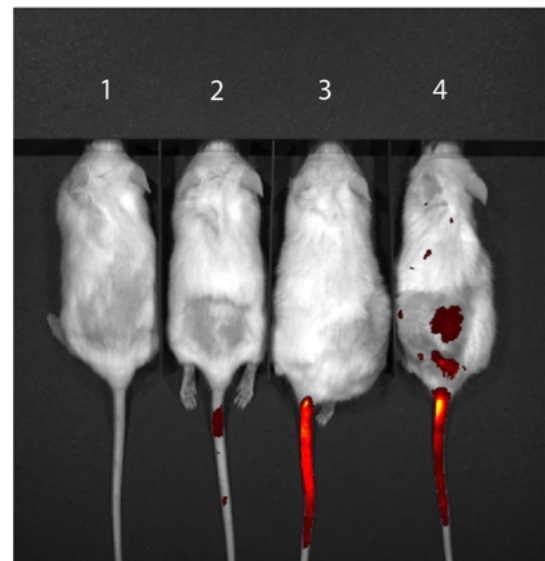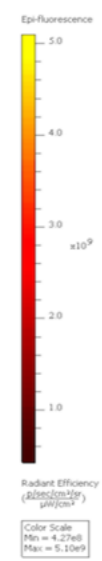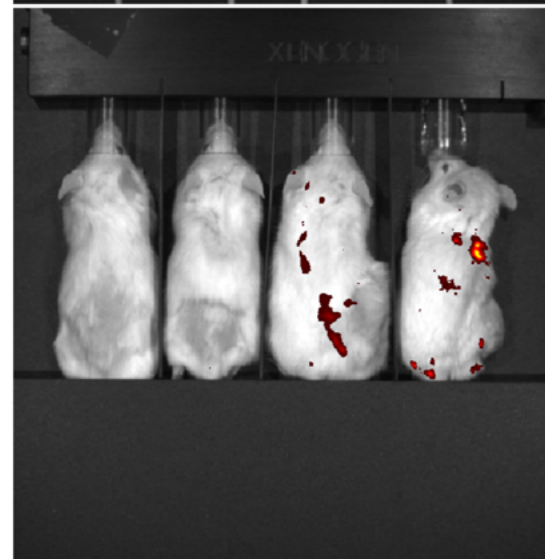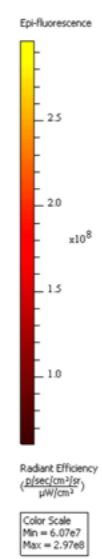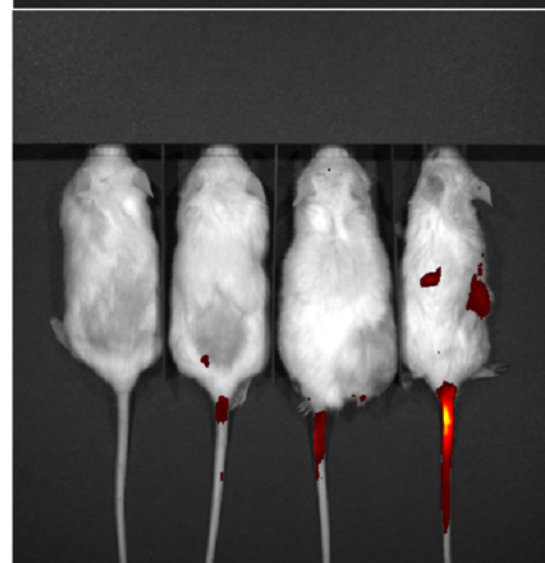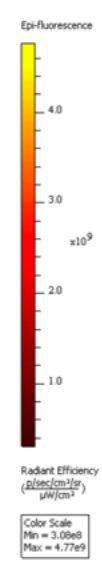

I

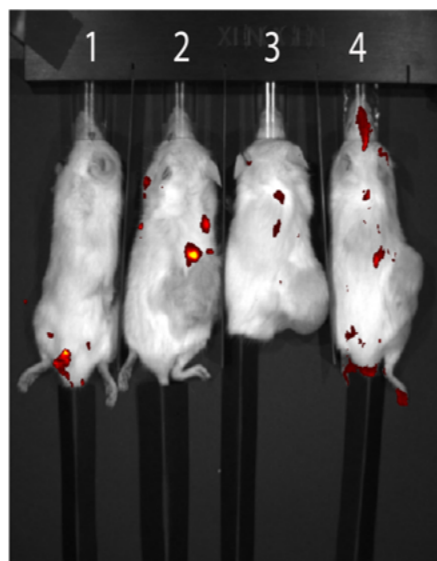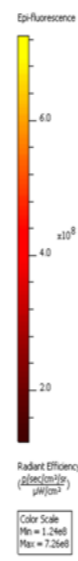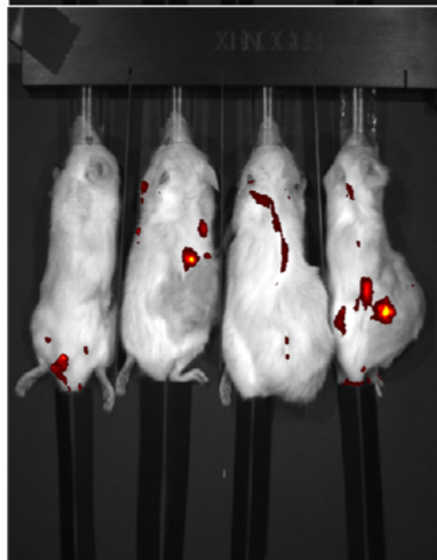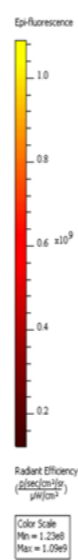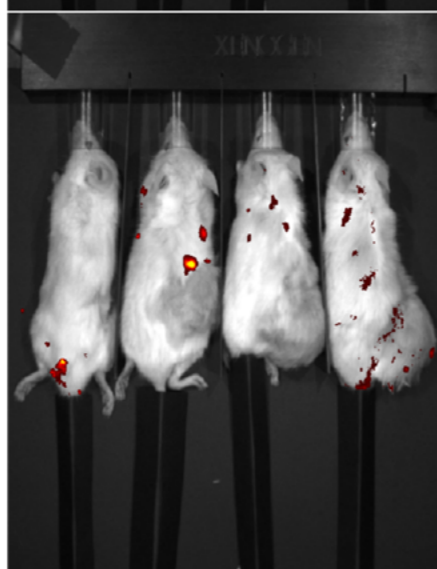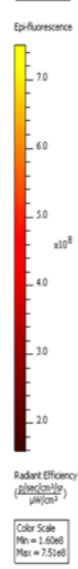

B

II

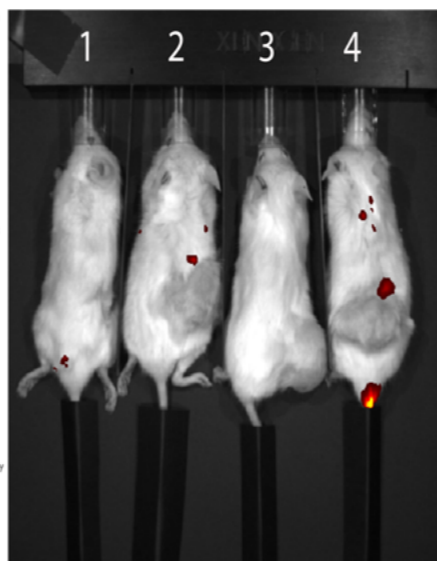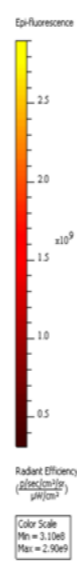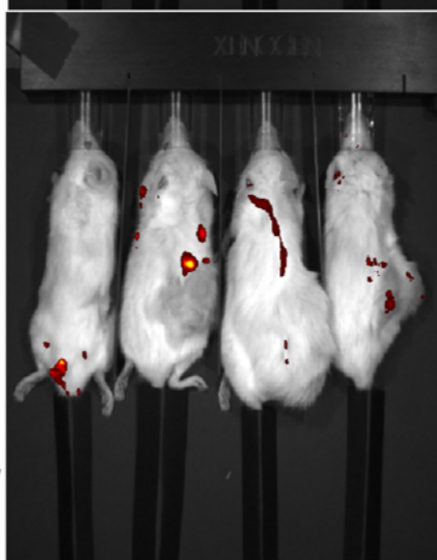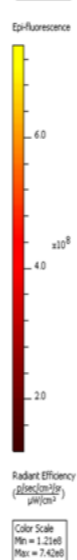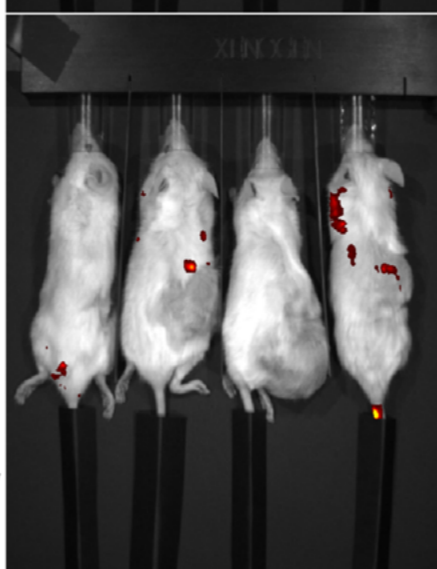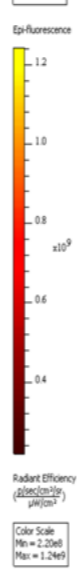

III

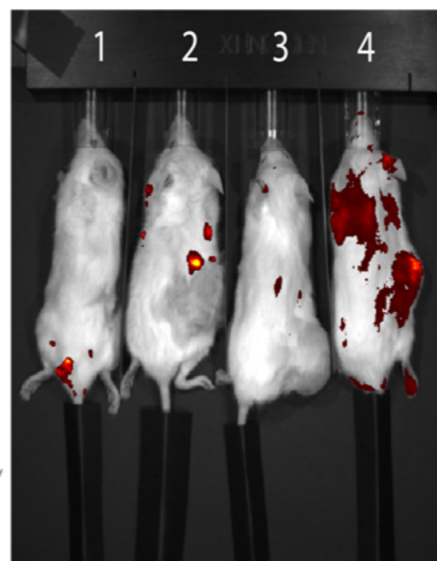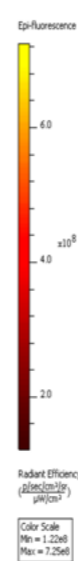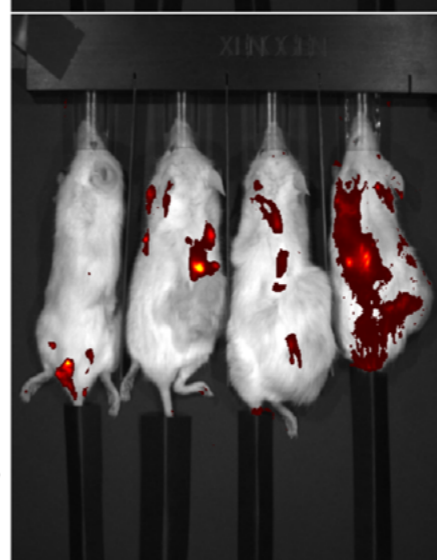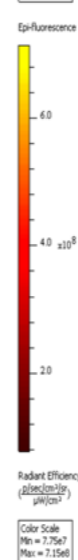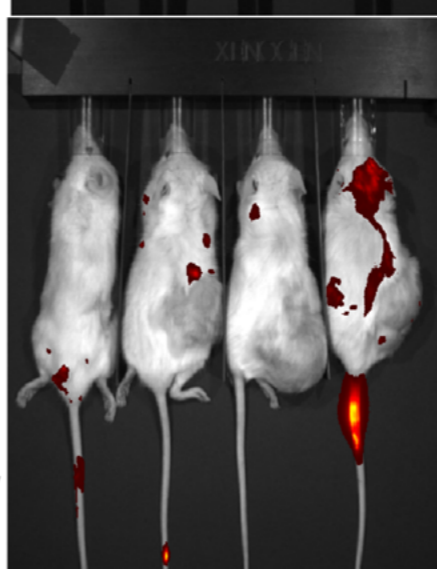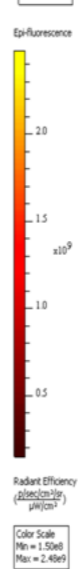

C

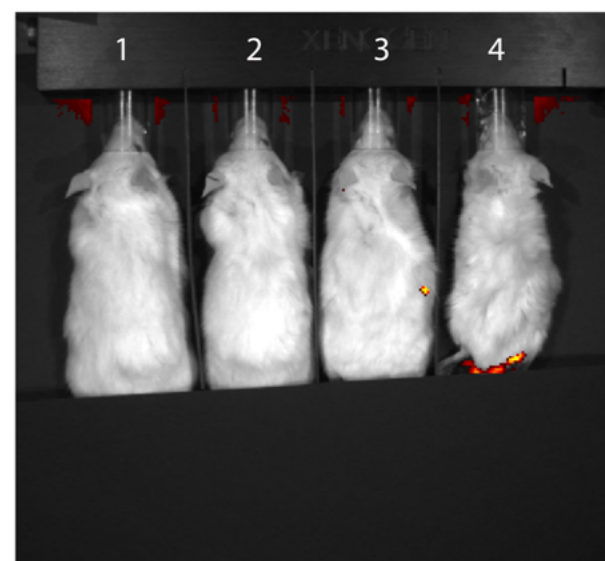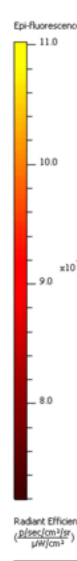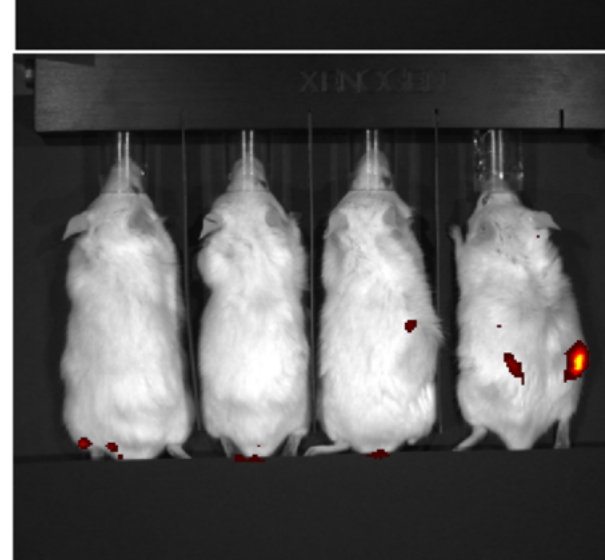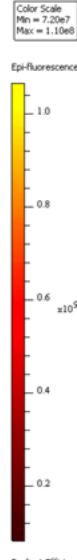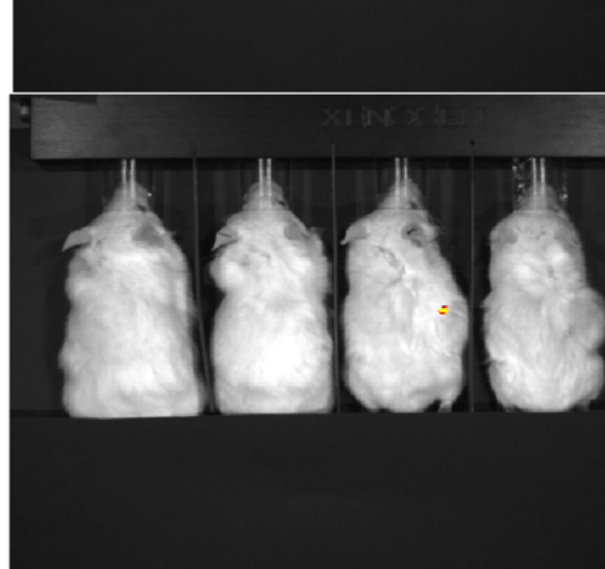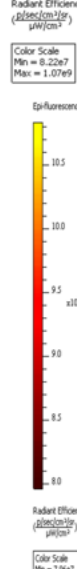

Supplement: Supplementary file 16 — Fig. S16. In vivo imaging in mice bearing PDX tumor models to detect apoptosis using a pan‐caspase assay done after the treatment course. The increased signal indicates higher caspase activity and apoptosis. A mouse with no tumor and no assay was used in each treatment group for comparing the signal. (A) TM01563 PDX Model. 1: A mouse with no tumor and with no pan‐caspase assay (One mouse for all cases) 2: A mouse with no tumor but receiving pan‐caspase assay (One mouse for all cases) 3: Three different mice from placebo treatment group receiving pan‐caspase assay 4: Three different mice from allopurinol treatment group receiving pan‐caspase assay. (B) TM0188 PDX Model. 1 (I, II, III): A mouse with no tumor and with no pan‐caspase assay (One mouse for all cases). 2 (I, II, III): A mouse with tumor and pan‐caspase assay but not getting any treatment (One mouse for all cases). 3 (I, II, III): Three different mice from placebo treatment group receiving pan‐caspase assay. 4 (I): Three different mice from allopurinol treatment group receiving pan‐caspase assay. 4 (II): Three different mice from CEP‐33779 treatment group receiving pan‐caspase assay. 4 (III): Three different mice from combination therapy group receiving pan‐caspase assay. (C) TM00206 PDX Model. 1: A mouse with no tumor and with no pan‐caspase assay (One mouse for all cases). 2: A mouse with tumor and pan‐caspase assay but receiving no treatment (One mouse for all cases) 3: Three different mice from placebo treatment group receiving pan‐caspase assay. 4: Three different mice from allopurinol treatment group receiving the pan‐caspase assay. [file MOL2-13-1725-s016.pdf]

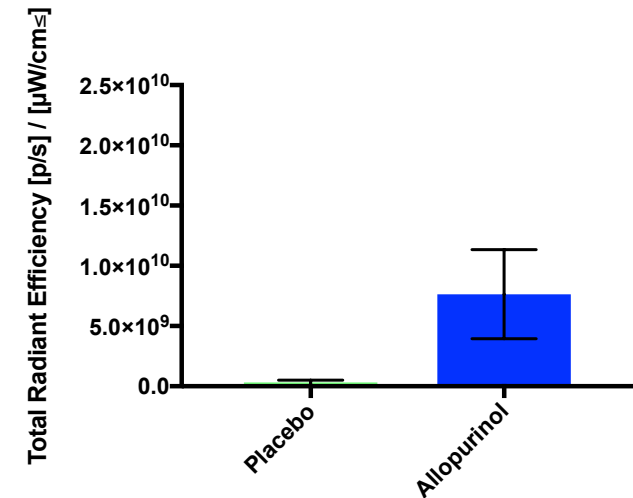

TM01563

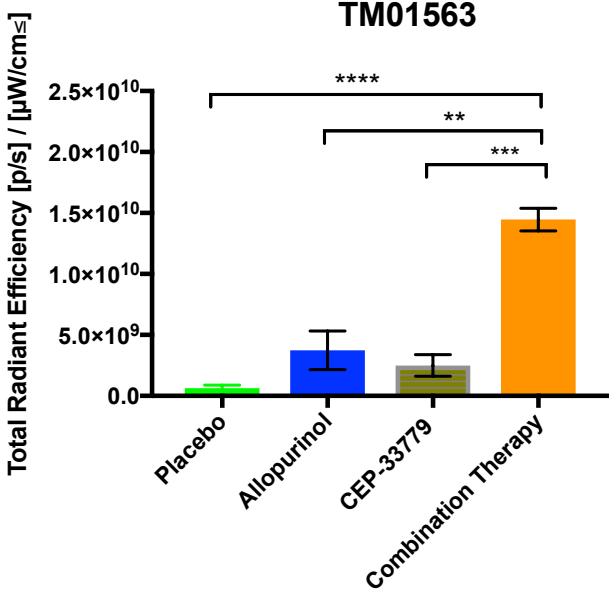

TM0188

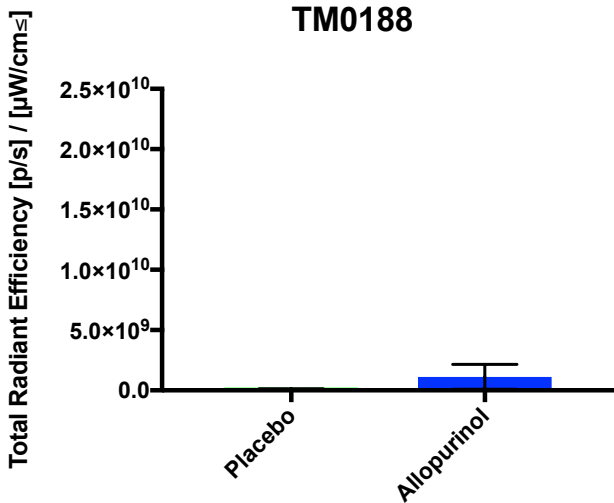

TM00206

Supplement: Supplementary file 17 — Fig. S17. Quantification of signals from pan‐caspase assay shown in Fig. S16. In this assay, a higher signal means a higher apoptosis rate. In each treatment group, the total radiant efficacy of the signal was compared to that from the mouse with no tumor and with no pan‐caspase assay. (mean + SEM, unpaired t‐test, **P < 0.01, ***P < 0.001). (Signals in foot pads and tails were not included in these plots). [file MOL2-13-1725-s017.pdf]

TM00206

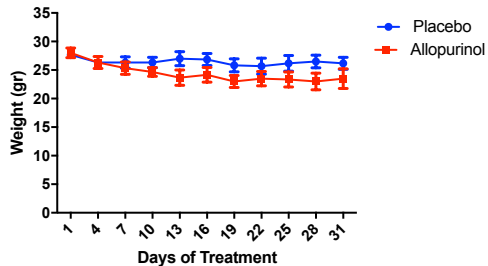

TM01563

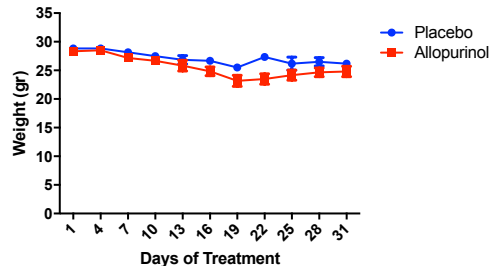

TM00188

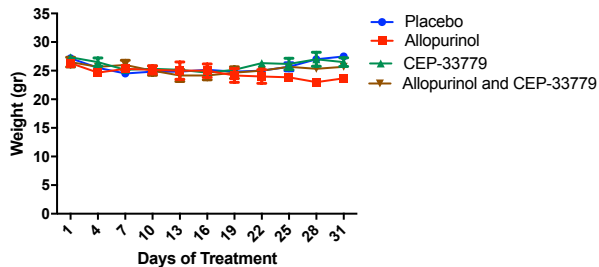

TM00939

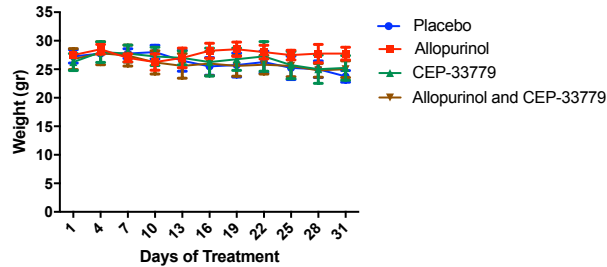

Supplement: Supplementary file 18 — Fig. S18. Changes in body weights for the mice used for in vivo study of PDX tumor models. [file MOL2-13-1725-s018.pdf]
