# Supplementary material for: Genomic signatures defining responsiveness to allopurinol and combination therapy for lung cancer identified by systems therapeutics analyses
Source: Mol Oncol. 2019 Jul 10;13(8):1725–43. doi: 10.1002/1878-0261.12521 (PMC6670022; doi:10.1002/1878-0261.12521)
Supplement: Supplementary file 10 — Fig. S10. Western blots showing the protein levels of JAK2 after siRNA knockdown. The JAK2 panels were cut from three different blots that included knockdown of other proteins not related to this communication and those bands have been removed. The GAPDH panels were run in one blot. [file MOL2-13-1725-s010.pdf]

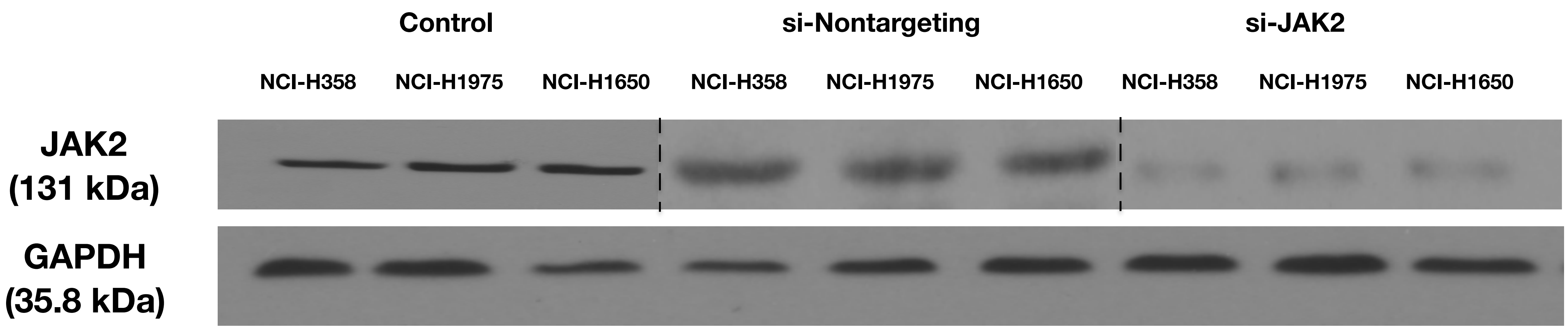
Control
si-Nontargeting
si-JAK2
NCI-H358
NCI-H1975
NCI-H1650
NCI-H358
NCI-H1975
NCI-H1650
NCI-H358
NCI-H1975
NCI-H1650
JAK2
(131 kDa)
GAPDH
(35.8 kDa)
